# Letermovir prophylaxis is effective in preventing cytomegalovirus reactivation after allogeneic hematopoietic cell transplantation: single-center real-world data

**DOI:** 10.1007/s00277-020-04362-2

**Published:** 2020-12-03

**Authors:** Patrick Derigs, Aleksandar Radujkovic, Maria-Luisa Schubert, Paul Schnitzler, Tilman Schöning, Carsten Müller-Tidow, Ute Hegenbart, Stefan O. Schönland, Thomas Luft, Peter Dreger, Michael Schmitt

**Affiliations:** 1 Department of Internal Medicine V (Hematology/Oncology/Rheumatology), Heidelberg University Hospital, Im Neuenheimer Feld 410, 69120 Heidelberg, Germany; 2 Center for Infectious Diseases, Virology, Heidelberg University Hospital, Heidelberg, Germany; 3 Department of Pharmacy, Heidelberg University Hospital, Heidelberg, Germany

**Keywords:** Letermovir, Cytomegalovirus, Real-world data, Hematopoietic-cell transplantation, Resource utilization

## Abstract

Morbidity and mortality after allogeneic hematopoietic cell transplantation (alloHCT) are still essentially affected by reactivation of cytomegalovirus (CMV). We evaluated 80 seropositive patients transplanted consecutively between March 2018 and March 2019 who received letermovir (LET) prophylaxis from engraftment until day +100 and retrospectively compared them with 80 patients without LET allografted between January 2017 and March 2018. The primary endpoint of this study was the cumulative incidence (CI) of clinically significant CMV infection (CS-CMVi) defined as CMV reactivation demanding preemptive treatment or CMV disease. With 14% CI of CS-CMVi at day +100 (11 events) was significantly lower in the LET cohort when compared to the control group (33 events, 41%; HR 0.29; *p* < 0.001). Whereas therapy with foscarnet could be completely avoided in the LET group, 7 out of 80 patients in the control cohort received foscarnet, resulting in 151 extra in-patient days for foscarnet administration (*p* = 0.002). One-year overall survival was 72% in the control arm vs 84% in the LET arm (HR 0.75 [95%CI 0.43–1.30]; *p* < 0.306). This study confirms efficacy and safety of LET for prophylaxis of CS-CMVi after alloHCT in a real-world setting, resulting in a significant patient benefit by reducing hospitalization needs and exposure to potentially toxic antiviral drugs for treatment of CMV reactivation.

## Introduction

Cytomegalovirus (CMV) reactivation contributes significantly to morbidity and mortality after allogeneic hematopoietic cell transplantation (alloHCT) [1–4]. As per current guidelines, the standard approach for preventing CMV-related complications is based on continuous monitoring of CMV viremia, triggering initiation of preemptive therapy (PET) upon detection of CMV reactivation [5, 6]. PET comprises antiviral agents like ganciclovir, valganciclovir, and foscarnet. However, these agents are associated with significant adverse effects such as myelo- or nephrotoxicity [7, 8], precluding their routine use for CMV prophylaxis. Accordingly, CMV viremia still represents a time-dependent risk factor for mortality after alloHCT [9].

Letermovir (LET) is an antiviral agent inhibiting CMV by binding to the components UL51 or UL56, or both, of the terminase complex that mediates CMV replication [10, 11]. In a pivotal phase 3 clinical trial, LET significantly reduced the incidence of clinically significant CMV infection (CS-CMVi) through week 24 after alloHCT when compared to placebo (18.9% vs 44.3%; *p* < 0.001) [12], leading to approval of LET for primary CMV prophylaxis in CMV-seropositive alloHCT recipients (CMV R+) in the European Union in January 2018. We adopted LET prophylaxis as standard policy in our institution as soon as the drug became commercially available in Germany in March 2018.

The aim of the present study was to investigate whether the positive results of the phase III trial could be reproduced under real-world conditions, and to analyze the impact of LET prophylaxis on the need for preemptive CMV therapy and CMV-related hospitalization.

## Methods

### Trial population and design

The study cohort consisted of the first seropositive 80 patients who received LET prophylaxis as standard of care and were transplanted at the Heidelberg University Hospital between March 2018 and March 2019. These were compared retrospectively with the last 80 seropositive patients transplanted prior to the implementation of LET in the institutional routine (January 2017–March 2018, control cohort). Primary endpoint was the cumulative incidence of CS-CMVi (CMV reactivation demanding PET, and/or CMV disease) at day +100 post alloHCT. All data were obtained by electronic chart review. Written informed consent for data analysis according to the Declaration of Helsinki was obtained from all patients. The local ethics committee had approved the sample and data collection and analysis.

### Definitions

Conditioning intensity was categorized according to the Working Group definitions [13]. For assignment of alloHCT risk, the EBMT score was applied [14].

### Procedures

Donor selection and conditioning was protocol-driven or followed institutional standard operating procedures in a JACIE-conform quality management environment [15]. GVHD prophylaxis and supportive care were performed as previously described [16].

CMV viremia was monitored by quantitative CMV PCR twice a week during the in-patient period and weekly thereafter. Throughout the entire study, plasma was used for detection of CMV DNAemia. Patients in the study received LET per os as primary prophylaxis 480 mg/day or 240 mg/day for patients being treated with cyclosporine. LET prophylaxis was given from engraftment until day +100 or CMV reactivation. Median time of beginning LET was 19 days after transplantation. If CMV viremia of more than 3200 IU/mL was detected, LET prophylaxis was stopped and systemic preemptive antiviral treatment was initiated. Standard therapy for CMV reactivation in out-patients and in-patients able to take the oral formulation of the drug was the administration of valganciclovir with doses of 450 mg twice daily orally for 14 days. If viremia was under control, a maintenance therapy with half the dose was pursued for a further 14 days. Aciclovir was stopped for the duration of preemptive therapy. In patients unable to take the oral formulation of the drug, ganciclovir was administered at a dose of 5 mg/kg body weight twice daily intravenously. Duration and maintenance therapy were according to the therapy with valganciclovir. If viremia was not under control after 2 weeks of therapy with valganciclovir or ganciclovir, preemptive therapy was changed to foscarnet. Foscarnet was administered with a dose of 90 mg/kg body weight twice daily intravenously until no CMV DNA was detectable in the patient’s blood. Doses were adjusted to the kidney function according to the prescribing information. Foscarnet could only be administered in an in-patient setting.

### DNA extraction and PCR amplification

DNA was extracted from 200 μL EDTA blood samples and purified using the QIAamp blood kit (QIAGEN, Hilden, Germany) according to the manufacturer’s instructions. A TaqMan real-time PCR assay was performed targeting the UL 86 region in the CMV genome. For quantitative analysis of CMV DNA, 5 μL of extracted nucleic acids was amplified with forward primer CMV1 (5’-CAG CCT ACC CGT ACC TTT CCA-3′) and reverse primer CMV2 (5’-GCG TTT AAT GTC GTC GCT CAA-3′) and detected with the probe 5’-FAM-TTC TAC TCA AAC CCC ACC ATC TGC GC-TAMRA-3′. Additionally, a CMV DNA quantification standard was used threefold in all assays in order to allow quantification of the amplified CMV DNA from patient samples. Quantified CMV DNA was expressed as IU/mL. PCR was performed in a reaction volume of 20 μL with a ready-to-use master mix (Roche Diagnostics, Mannheim, Germany) containing Taq DNA polymerase and dNTPs. Amplification and detection were performed on a LightCycler 480 instrument (Roche Diagnostics, Mannheim, Germany) with a thermocycling profile at 95 °C for 5 min followed by 50 cycles of 95 °C for 5 s and 60 °C for 20 s.

### Statistical methods

Survival curves for overall survival were estimated by the Kaplan-Meier method and compared between groups using the log-rank test. Taking death into account as competing risk, cumulative incidence curves were estimated for CS-CMVi and compared between groups using Gray’s test [17]. Patients reactivating CMV prior to engraftment were not considered event in both groups. Categorical variables were described by absolute and relative frequencies. Fisher’s exact test or the chi-square test was used to compare categorical factors between groups of patients. For continuous variables, the Mann-Whitney *U* test was applied. Survival times calculations were done using GraphPad Prism software (release 5.2; San Diego, CA, USA), IBM SPPS Statistics (release 24.0; Armonk, NY, USA), and the statistical software environment R (release 3.3.2; Vienna, Austria), together with the R packages ‘maxstat’ (release 0.7-25), ‘knitr’ (release 1.20), ‘survplot’ (release 0.0.7), ‘rms’ (release 5.1-2), ‘cmprsk’ (release 2.2–7), and ‘survival’ (release 2.42-6). Significance levels were set at 0.05. Data were analyzed as of April 30, 2020.

## Results

### Patients

Whereas the study cohort tended to contain more female patients than the control, both cohorts were comparable in terms of age, diagnosis, donor source, conditioning intensity, use of ATG, pharmacological immunosuppression, performance status, and alloHCT risk. Of note, both groups were fully matched for CMV donor/recipient sero-status. Patient characteristics are detailed in Table 1.

**Table 1:** Patient characteristics

Characteristics	Letermovir Group (*N* = 80)	Control group (*N* = 80)	*p*
Age—years			0.497
Median	58.5	58.0	
Range	18–75	28–70	
Male sex—no. (%)	51 (64)	39 (49)	0.080
CMV-status donor/recipient—no. (%)			1.000
Positive/positive	62 (78)	62 (78)	
Negative/positive	18 (23)	18 (23)	
Diagnosis—no. (%)			0.796
Acute myeloid leukemia	31 (39)	34 (43)	
MPS/MDS	23 (29)	19 (24)	
Lymphoma	15 (19)	13 (16)	
Other disease	11 (14)	14 (18)	
HLA matching and donor type—no. (%)			0.750
Matched unrelated	45 (56)	40 (50)	
Matched related	21 (26)	19 (24)	
Mismatched related	1 (1)	1 (1)	
Haploidentical related	3 (4)	4 (5)	
Mismatched unrelated	10 (13)	16 (20)	
Conditioning regimen^a^—no. (%)			0.718
Myeloablative	19 (24)	22 (28)	
Reduced-intensity	61 (76)	58 (73)	
Antithymocyte globulin use—no. (%)	57 (71)	55 (69)	0.863
Immunosuppressant use—no. (%)			0.565
Cyclosporine/methotrexate	58 (73)	65 (81)	
Cyclosporine/mycophenolate mofetil	11 (14)	11 (14)	
Tacrolimus/methotrexate	5 (6)	2 (3)	
Tacrolimus/mycophenolate mofetil	6 (8)	2 (3)	
Performance status at alloHCT^b^—no. (%)			0.402
≤ 80%	5 (6)	9 (11)	
90–100%	75 (94)	71 (89)	
AlloHCT risk (EBMT score)^c^—no. (%)			0.970
0–2	11 (14)	10 (13)	
3–4	38 (48)	39 (49)	
5–7	31 (39)	31 (39)	

*N*, number; *CMV*, cytomegalovirus; *MDS*, myelodysplastic syndrome; *MPS*, myeloproliferative syndrome; *alloHCT*, allogeneic hematopoietic cell transplantation; *EBMT*, European Group for Blood and Marrow Transplantation

^a^According to Bornhäuser et al. [18] and Bacigalupo et al. [13]

^b^According to Karnofsky et al. [19]

^c^According to Gratwohl et al. [14]

### Outcome

**Fig. 1:**
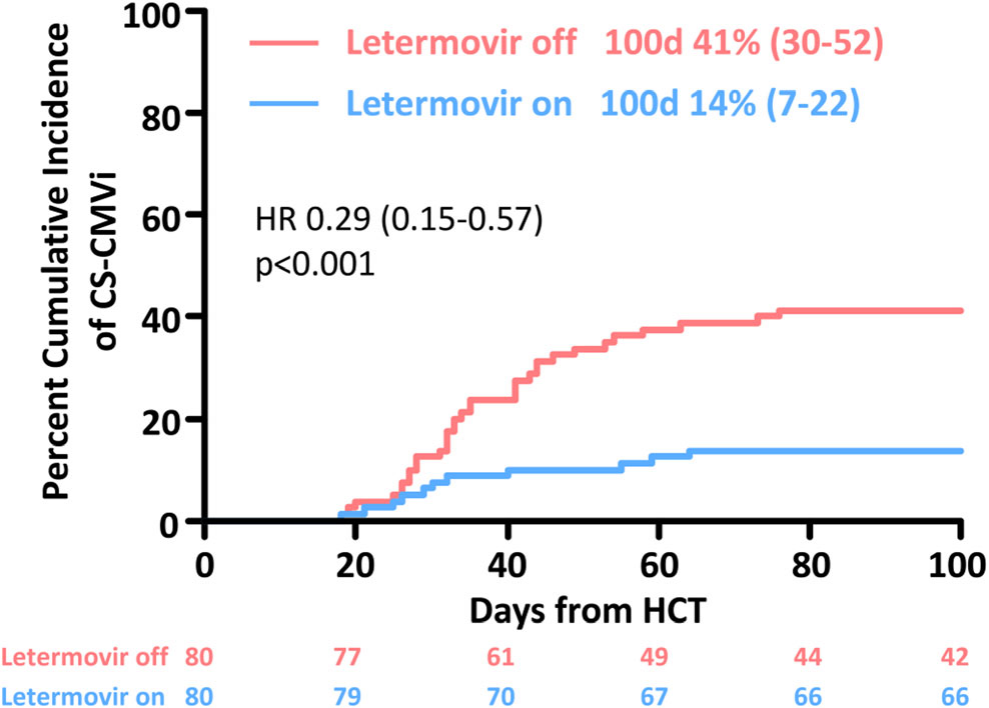
Cumulative incidence of clinically significant cytomegalovirus infection (CS-CMVi) through day 100 post allogeneic hematopoietic cell transplantation (HCT) in patients with (letermovir on) and without (letermovir off) letermovir prophylaxis

With altogether 11 reactivation events, the cumulative incidence of CS-CMVi on day +100 was 14% (95%CI 7–22%) in the LET cohort which was significantly lower than in the control group (33 events, day +100 cumulative incidence 41% (95%CI 30–52%); HR 0.29 (95%CI 0.15–0.57); *p* < 0.001), as shown in Fig. 1. Consequently, LET prophylaxis was associated with 3.5-fold lower risk of CS-CMVi within the first 100 days post-transplant. The median peak CMV viral load of patients with CS-CMVi was comparable in both groups (median 13,654 IU/mL in the LET cohort vs median 19,357 IU/mL in the control cohort; *p* = 0.540; Mann-Whitney *U* test). The median time to CMV reactivation of patients with CS-CMVi was 35 days in the LET cohort compared to 38 days in the control group (*p* = 0.369; Mann-Whitney *U* test).

Four out of 80 patients in the control cohort reactivated with CMV DNAemia prior to engraftment when compared to two out of 80 patients in the LET cohort. Only one patient in the control arm received PET prior to engraftment. The other 3 patients did not receive PET due to self-limiting minor CMV reactivations. In the LET arm, one patient did not receive PET after reactivating CMV prior to engraftment due to minor and self-limiting CMV DNAemia. The other patient was treated with foscarnet from day 13 until day 28 after transplantation. PET was stopped due to renal impairment and LET prophylaxis was started on day 34 when the patient was CMV DNA negative. There was no second CMV reactivation recorded under LET prophylaxis in this patient.

The lower incidence of CMV reactivation associated with LET prophylaxis was mirrored by a significant reduction of need for preemptive CMV treatment (10 and 28 episodes in the LET and control arms, respectively; *p* = 0.001; Fisher’s exact test). Whereas therapy with foscarnet could be completely avoided in the LET group, 7 out of 80 patients in the control cohort received foscarnet, resulting in 151 extra in-patient days for foscarnet administration apart from initial hospitalization for alloHCT (*p* = 0.014; Fisher’s exact test). The cumulative number of days on valganciclovir before day +100 was 368 for LET patients vs 836 for control patients (*p* = 0.002; Mann-Whitney *U* test).

While seven deaths occurred within the first 100 days post-transplant in the control arm, the number of deaths in the LET arm was four, resulting in a 100-day overall survival (OS) of 91.3% and 96.3%, respectively. In the LET arm, three patients died due to non-relapse mortality (NRM) and one patient died because of progressive disease (PD). In the control arm, five patients deceased due to NRM and two due to PD. Two patients of the control cohort developed CMV end-organ disease in the form of CMV-associated colitis. No patient in the LET group developed CMV end-organ disease (*p* = 0.497; Fisher’s exact test). There were no adverse events related to LET prophylaxis observed in the LET cohort.

**Fig. 2:**
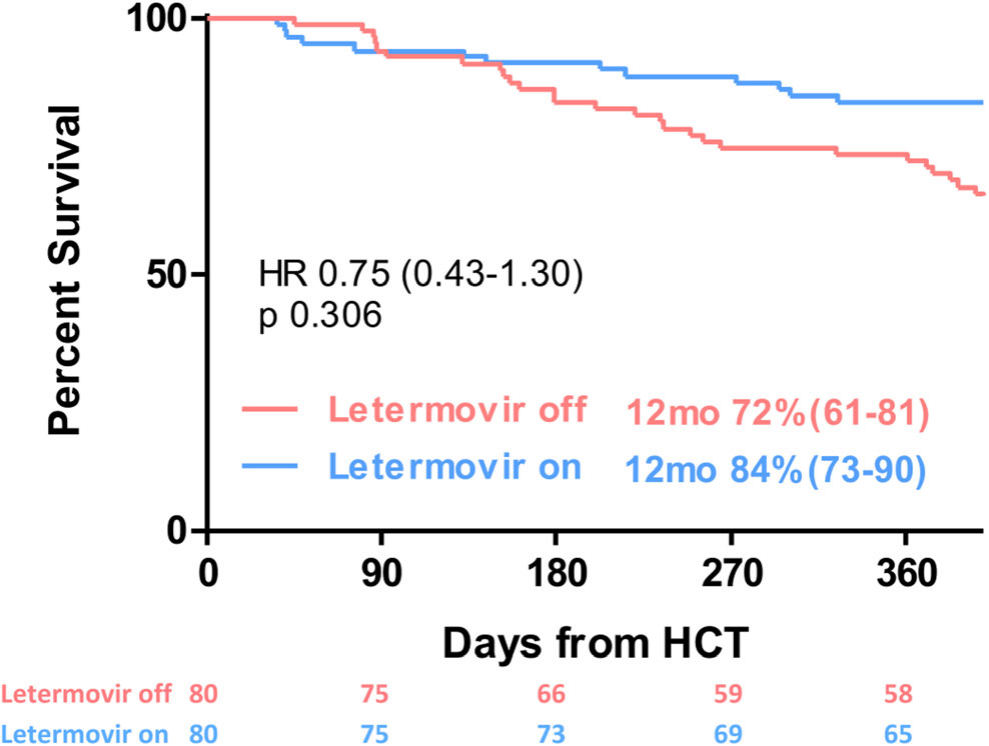
Overall survival within 1 year post allogeneic hematopoietic cell transplantation (HCT) of patients with (letermovir on) and without (letermovir off) letermovir prophylaxis

**Fig. 3:**
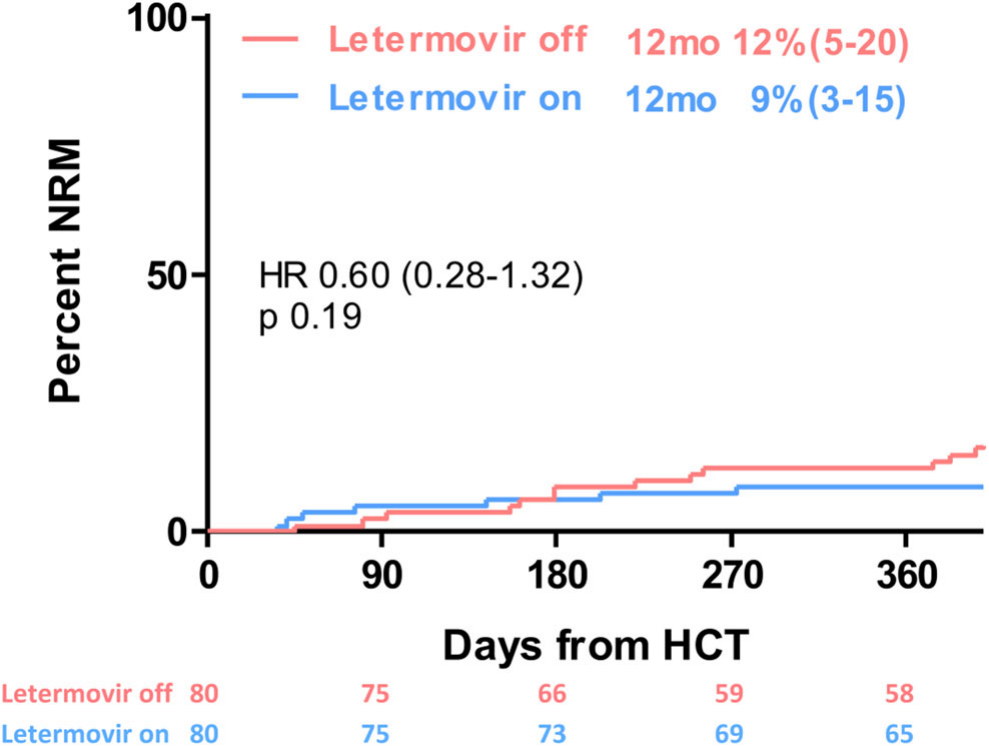
Non-relapse mortality (NRM) within 1 year post allogeneic hematopoietic cell transplantation (HCT) of patients with (letermovir on) and without (letermovir off) letermovir prophylaxis

One-year OS was 72% in the control arm vs 84% in the LET arm (HR 0.75 [95%CI 0.43–1.30]; *p* < 0.306), as shown in Fig. 2. The cumulative risk of NRM at 1 year post-transplant was 12% in the control cohort compared to 9% in the LET cohort (HR 0.60 [95%CI 0.28–1.32]; *p* < 0.190; Fig. 3).

## Discussion

Although CMV management in the alloHCT setting has largely improved over the past decades due to the advent of effective CMV-targeting drugs, such as foscarnet and ganciclovir, along with the introduction of sensitive CMV monitoring, CMV still contributes significantly to morbidity and mortality after alloHCT [20]. Since foscarnet and ganciclovir are unsuitable for prophylactic use in allograft recipients because of toxicity [21, 22], a variety of novel anti-viral compounds have been explored for post alloHCT prophylaxis [12, 23, 24]. Of these, LET was the first agent receiving FDA and EMA approval for this indication.

With LET, a new drug was introduced for CMV prophylaxis that eventually did not show any relevant adverse effects. In a pivotal phase 3 clinical trial, its safety and effectiveness were demonstrated impressively [12]. Furthermore, all-cause mortality was reduced by LET at 24 weeks after alloHCT [25]. Of note, neither myelosuppression nor nephrotoxicity was observed in patients under LET prophylaxis, which is a major advantage of LET as compared to (val-)ganciclovir and foscarnet.

Here, we conducted a retrospective study to evaluate the benefits of LET prophylaxis if used as standard of care in a real-world setting. In contrast to the approval trial, where LET prophylaxis was started a median of 9 days after alloHCT and administered through week 14 (approximately day 100 after transplantation), we started LET prophylaxis only after stable neutrophil engraftment which was achieved in the third week post-transplant in the majority of patients. Therefore, the median LET prophylaxis starting time point in our study was 10 days later than in the phase 3 clinical trial. This might at least partially explain the difference between the cumulative incidences of CS-CMVi by day 100 after transplantation of our study and of the pivotal trial. With 14% in the LET cohort, the incidence was higher than in the phase 3 clinical trial (8% in the LET group). The incidence of the control group was comparable (41% in our study as compared to 39% in the phase 3 clinical trial).

The median time to CMV reactivation of patients with CS-CMVi was similar in both groups. This suggests that there might not be any effect of LET on CMV replication in patients reactivating CMV under LET prophylaxis. Resistance against LET seems to be present directly after transplantation and patients reactivate in the same way than those in the control group.

In keeping with the approval trial, in the present study, the safety profile of LET was generally excellent. Notably, there was no case of discontinuation of LET prophylaxis due to adverse effects in our study.

Similar to the approval trial, in the present study, LET resulted in a significant reduction of the proportion of patients needing PET (approval trial, 7% vs 38% in the control arm; our study, 14% vs 41% in the control cohort). Specifically, hospitalization for foscarnet administration and the total number of patient days on valganciclovir were decreased significantly by LET in the present analysis. While this study was not designed to evaluate the economic impact of LET prophylaxis by weighing LET costs (about 200–400 EUR per day depending on the type of immunosuppression used) against PET, it is obvious that less hospitalization and less exposure to potentially toxic drugs represent a clinical benefit for the patients.

The introduction of LET as a universal prophylaxis against CMV reactivation after alloHCT enables physicians to pursue a more personalized way of treating breakthrough CMV reactivations. LET presents a first strong hurdle that has to be taken by CMV in order to lead to CMV disease. Besides the patient’s individual risk profile for CMV disease as an independent rule for CMV vulnerability, more individual parameters are getting important to find the right therapy of CMV reactivations. It is important to consider parameters measured at the time of initiation of preemptive antiviral therapy like CMV-specific T cell response and CMV viral load. In the future, detection of drug resistance will be of great importance [26].

In conclusion, the clinical benefit of LET prophylaxis suggested by the approval trial could be reproduced in a real-world setting and implicated a significant reduction of hospitalization for foscarnet and days on valganciclovir. While this is a benefit per se in terms of patient safety and convenience, it remains to be proven if LET can impact the excess mortality still associated with CMV positivity of alloHCT recipients.

## Data Availability

The datasets generated during and/or analyzed during the current study are available from the corresponding author on reasonable request.
